# Numerical Analysis of Laser Thermal Ablation for Gingival and Peri‐Implant Inflammation Using Various Laser Irradiation Angles

**DOI:** 10.1002/cnm.70080

**Published:** 2025-09-12

**Authors:** Donghyuk Kim, Hyunjung Kim, Hee‐Sun Kim

**Affiliations:** ^1^ Department of Mechanical Engineering Ajou University Suwon‐si Gyeonggi‐do Korea; ^2^ Department of Dentistry SMG_SNU Boramae Medical Center Seoul Korea

**Keywords:** Arrhenius thermal damage ratio, dental implants, numerical analysis, Peri‐implantitis, photothermal therapy

## Abstract

Because of tooth decay and loss, many people use dental implants as tooth replacements. However, careless management can trigger an inflammatory reaction to an implant. Recently, various studies have been conducted on the sterilization and purification of implant surfaces using diodes, Er:YAG lasers, and CO_2_ lasers. In this study, the therapeutic effect of photothermal therapy, a laser treatment method for peri‐implantitis, was analyzed through numerical analysis based on heat‐transfer theory. The numerical simulation conditions included laser power values in the range of 0.0–4.0 W, laser irradiation angles ranging from 15° to 40°, and laser irradiation durations of 100–500 s. In addition, the Arrhenius damage integral was used to quantitatively verify the therapeutic effects of the photothermal therapy. An analysis of the results confirmed that the Arrhenius thermal damage ratio and normal tissue Arrhenius thermal damage ratio decreased as the laser irradiation angle increased. However, for the same normal tissue Arrhenius thermal damage ratio, the effective laser intensity increased with the laser angle. Finally, the effectiveness of photothermal therapy for peri‐implantitis under various conditions was confirmed. These results are expected to optimize the clinical treatment of peri‐implantitis in the future.

## Graphical abstract

In this study, the therapeutic effect of photothermal therapy was analyzed through numerical analysis. The Arrhenius damage integral was used to quantitatively verify the therapeutic effects. An analysis of the results confirmed that the Arrhenius thermal damage ratio and normal tissue Arrhenius thermal damage ratio decreased as the laser irradiation angle increased.

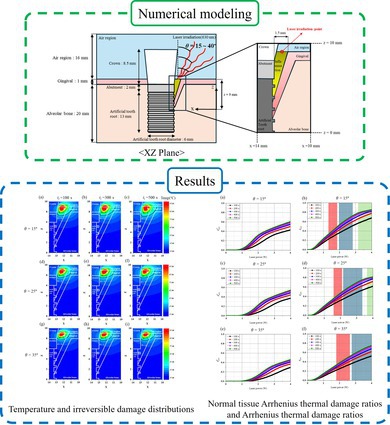

## 1 Introduction

A dental implant compensates for the loss of a normal tooth. Proper management is necessary for long‐term implant maintenance, as peri‐implantitis may develop 5–10 years after placement due to neglect [1, 2]. Peri‐implantitis arises from plaque formed by food residue, drinking, and smoking, which accumulates as a bacterial film on the implant surface [3, 4]. This plaque and bacterial film increase the gap between the implant and gums, leading to gum tissue inflammation [5, 6]. Diagnosis of peri‐implantitis occurs when probe depth exceeds 4 mm. Untreated, it can damage surrounding gum and bone tissue, necessitating implant removal in severe cases. Implant removal and reimplantation take at least 6 months and are costly [7, 8].

Methods for managing peri‐implantitis include physical removal with brushes and curettes, local or systemic antibiotics, and topical disinfectants [9, 10]. However, physical removal methods may harm implant surfaces and surrounding tissues, particularly in deeply inflamed areas. In addition, antibiotics and disinfectants can cause side effects or may not yield immediate results [11]. Laser treatment methods have gained considerable attention for overcoming these limitations. Compared to traditional methods, lasers cause less bleeding, post‐procedural pain, and effectively reduce inflammation, thereby reducing patient psychological burden [12, 13].

Photothermal therapy (PTT) utilizes laser treatment to convert light into heat energy, effectively reducing inflammation by raising tissue temperature, quantified through the Arrhenius damage integral [14, 15, 16]. PTT is being studied not only in inflammation, but also in medicine and bacterial disinfection [17, 18, 19, 20]. However, high laser intensities and long irradiation times can potentially damage surrounding normal tissues, as indicated by the Arrhenius damage integral. Therefore, in dental laser treatments, minimizing normal tissue damage while maximizing inflammation relief is crucial [21, 22].

Recent research focuses on measuring temperature changes on implant surfaces under various laser conditions. For instance, Deppe et al. [23] used a 445 nm diode laser to assess temperature changes on the tops of five different implant types using six different irradiation methods. They observed and compared temperature changes before and after laser exposure, identifying implants with the largest and smallest temperature fluctuations under each condition. Similarly, Fahlstedt et al. [24] studied temperature variations in titanium implants with three surface treatments. They employed a dual‐wavelength laser system combining a 940 nm diode laser and a 2780 nm Er, Cr:YSGG laser, along with a water/air spray for implant cooling during approximately 3 min of irradiation. Experimental results showed that implant surface temperatures decreased to approximately 26°C from an initial 37°C, highlighting the effective cooling achieved by the water/air spray, which offset the heating effects of the dual‐wavelength laser.

A review of the literature revealed that most studies only measured the surface temperature changes of implants, without quantifying thermal damage to the inflamed or surrounding tissue. Therefore, in this study, we aimed to determine the temperature distributions in the inflamed and surrounding tissues during peri‐implantitis treatment using photothermal therapy under various laser intensities, angles, and irradiation times. The degree of inflammation and irreversible damage to normal tissues, based on temperature distribution, was quantified using the Arrhenius damage integral, defining the range of cell death for each condition. Finally, the therapeutic effect of photothermal therapy was confirmed by calculating the ratio of the volume where the Arrhenius damage integral exceeded one to the total volume of normal tissue and inflammation. This study builds upon the work of Kim et al. [25] by additionally calculating the time‐dependent temperature distribution within inflamed or surrounding tissue. The establishment of a model for calculating the transient temperature field addressed in this study is considered innovative, as it provides a method for predicting the temperature distribution in peri‐implantitis. It is expected to lay the groundwork and serve as a guide for future numerical studies on peri‐implantitis.

## 2 Theory and Methods

### 2.1 Heat Transfer Model

In this study, the temperatures of the laser‐irradiated peri‐implantitis and surrounding tissues were calculated using the Pennes bioheat equation [26]. This equation includes a heat transfer term due to blood flow and a heat generation term due to metabolism, in addition to the existing thermal diffusion equation. Since this study utilized a laser to apply heat, a term for the heat generated by the laser was also included. The final form of the equation is shown in Equation (1).

(1) $$\rho c_{p}\frac{\partial T}{\partial t}=k\nabla^{2}T+q_{b}+q_{m}+q_{l}$$

(2) $$q_{b}=\rho_{b}\omega_{b}c_{p,b}\left(T_{b}-T\right)$$

(3) $$q_{l}=\left(1-R_{t}\right)\cdot \mu_{a}\frac{P_{l}\cdot \text{cosθ}}{\pi r_{l}^{2}}e^{-\mu_{\mathrm{tot}}\left(z^{\prime }\right)}\cdot e^{-\frac{{\left(x^{\prime }\right)}^{2}+y^{2}}{r_{l}^{2}}},\left(\mu_{\mathrm{tot}}=\mu_{a}+\mu_{s}^{\prime }\right),\mu_{s}^{\prime }=\mu_{s}\left(1-g\right)$$

(4) $$\left(\begin{array}{c} x^{\prime }\\ z^{\prime } \end{array}\right)=\left(\begin{array}{cc} \text{cosθ} & -\text{sinθ}\\ \text{sinθ} & \text{cosθ} \end{array}\right)\left(\begin{array}{c} x\\ z \end{array}\right)$$

Here, *ρ*, *c*_*p*_, and *k* represent the density, specific heat, and thermal conductivity, respectively. The terms *q*_*b*_, *q*_*m*_, and *q*_*l*_ are the heat transfer values due to blood, the metabolic heat source, and the laser heat source, respectively. *q*_*m*_ is treated as a constant, and *q*_*b*_ and *q*_*l*_ are expressed by Equations (2) and (3), respectively. In Equation (2), *ρ*_*b*_, *ω*_*b*_, *c*_*p,b*_, and *T*_*b*_ represent blood density, blood perfusion rate, blood specific heat, and blood temperature, respectively. The wavelength of the laser used in this study is 630 nm, a type of diode laser. The parameters *R*_*t*_, *μ*_*a*_, *P*_*l*_, *r*_*l*_, *θ*, and *μ*_*tot*_ in Equation (3) represent total reflectivity, absorption coefficient, laser power, laser radius, laser irradiation angle, and attenuation coefficient, respectively. Laser energy transfer in a three‐dimensional situation was applied through Equation (3). $e^{-\mu_{\mathrm{tot}}z^{\prime }}$ term in Equation (3) reflects the energy attenuated in the depth direction, and $e^{-\frac{{\left(x^{\prime }\right)}^{2}+y^{2}}{r_{l}^{2}}}$ term reflects the energy attenuated in the radial direction. Furthermore, for energy transfer due to the change of laser angle, the rotational matrix is applied as shown in Equation (4). Since it is a rotation conversion in the *x* and *z* coordinates, changes were applied to the *x* and *z* terms. Additionally, *dx* and *dz* are differential lengths used to determine the laser irradiation position, and *μ*_*tot*_ can be expressed as the sum of *μ*_*a*_ and the reduced scattering coefficient, *μ'*_*s*_ [27, 28].

(5) $$R_{t}=R_{1}+R_{2},R_{1}={\left[\frac{\sqrt{n_{2}^{2}-{\left(n_{1}\text{sinθ}\right)}^{2}}-n_{1}\text{cosθ}}{\sqrt{n_{2}^{2}-{\left(n_{1}\text{sinθ}\right)}^{2}}+n_{1}\text{cosθ}}\right]}^{2}$$

Here, *R*_*t*_, representing the total reflectivity of the laser, is expressed as the sum of the specular reflectivity, *R*_*1*_, and the diffuse reflectivity, *R*_*2*_. The *R*_*2*_ value of the inflammation is 0.28 at the wavelength of the 630 nm laser Equation (5) [29]. The *R*_*1*_ value is determined by the angle *θ* at which the laser is irradiated, along with the refractive indices *n*_*1*_ for air (= 1) and *n*_*2*_ for the inflammation (= 1.373) [27, 30].

### 2.2 Arrhenius Variable

To determine the degree and extent of any irreversible damage to the inflamed and normal tissues, this study used the Arrhenius damage integral expressed in Equation (6) [31]. Here, the Arrhenius damage integral value (Ω) represents the degree of damage; *A* (= 2.84 × 10^99^ s^−1^) is the frequency factor, which is the probability that the reaction will occur; *E*_*a*_ (= 6.19 × 10^5^ J/mol) is the activation energy, the minimum energy required for the reaction to start; *R* (= 8.314 J/(mol·K)) is the ideal gas constant; and *T*(*t*) is the temperature at time *t* [32]. If Ω exceeded 1, irreversible damage was considered to have occurred [33, 34].

(6) $$\Omega \left(t\right)=\int_{0}^{t}Ae^{-\frac{E_{a}}{\mathrm{RT}\left(t\right)}}\;\mathrm{dt}$$

After calculating Ω for each grid in both the inflamed and normal tissues, the Arrhenius variable proposed by Paik et al. [35] was used to quantify the degree of irreversible damage. Here, *ϕ*_*Arrh*_ is the Arrhenius thermal damage ratio, which represents the ratio of the volume of inflammation with Ω > 1 to the total volume of the inflammation Equation (7); and *ϕ* ^*N*^_*Arrh*_ is the normal tissue Arrhenius thermal damage ratio, which represents the ratio of the volume of normal tissue with Ω > 1 to the total volume of normal tissue Equation (8). The normal tissue range was set to 50% of the inflammation transverse length [36].

(7) $$\phi_{\text{Arrh}}=\frac{\text{Inflammation Volume}\;\mathrm{at}\;\Omega \left(t\right)>1}{\text{Total Inflammation Volume}}$$

(8) $$\phi_{\text{Arrh}}^{N}=\frac{\text{Normal tissue Volume}\;\mathrm{at}\;\Omega \left(t\right)>1}{\text{Total Normal tissue Volume}}$$

### 2.3 Numerical Investigation

**FIGURE 1:**
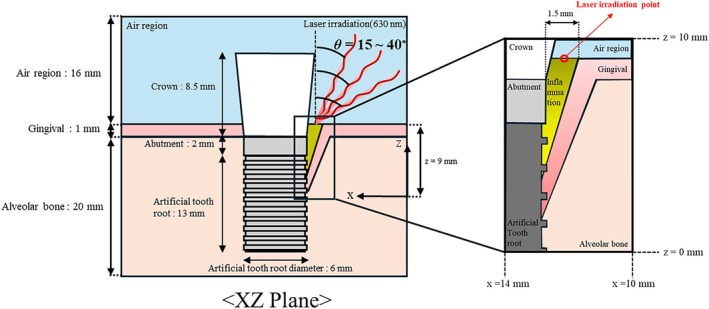
Schematic of numerical model.

This study used numerical analysis to simulate photothermal therapy for inflammation occurring between implants and gums. Figure 1 shows a view from the XZ plane of a three‐dimensional numerical model, illustrating inflammation situated between the gum and implant in the form of a cone, measuring 1.5 mm in transverse length and 9 mm in height. The heights of the crown, abutment, and artificial tooth root were set to 8.5, 2, and 13 mm, respectively, and the diameters of the artificial tooth root and abutment were set to 6 mm. For the crown, a truncated cone shape was set, with a diameter of 6 mm at the bottom and 7.49 mm at the top, and 15° on the side of the crown. The height of the air region, gingival, and alveolar bone surrounding the implant was set to 16, 1, and 20 mm, respectively. The thermal and optical properties of these components are listed in Table 1.

**TABLE 1:** Thermal and optical properties of implant and surroundings [31, 37, 38, 39, 40, 41, 42, 43, 44, 45].

	*k* (W/(m·K))	*c*_*p*_ (J/(kg·K))	*ρ* (kg/m^3^)	*ω*_*b*_ (1/s)	*q*_*m*_ (W/m^3^)	*μ*_*a*_ (1/cm)	*μ'*_*s*_ (1/cm)
Inflammation	0.48	3500	1080	0.009	65,400	1.87	8.4
Gingiva	0.63	4200	1000	0.0076	1091	0.53	3.817
Alveolar bone	0.38	1260	2060	0.00369	—	0.596	22.97
Crown (zirconia)	2.80	450	6080	—	—	0.10	20.43
Abutment (zirconia)	2.80	450	6080	—	—	0.10	20.43
Artificial tooth root (Ti‐6Al‐4 V)	7.00	546	4420	—	—	789,500	≈ 0
Air	0.0256	1.006	1.205	—	—	0	0
Blood	—	4200	1000	—	—	—	—

Table 2 summarizes the numerical analysis conditions. Numerical analyses were performed for a total of 3535 cases. The laser power (*P*_*l*_) was set at values in the range of 0.0–4.0 W in 0.04 W steps, the laser irradiation angle (*θ*) was varied from 15° to 40° in 5° steps, and the laser irradiation time (*t*_*l*_) ranged from 100 to 500 s in 100 s steps. The minimum angle was set to 15° because the angle of the side of the crown is generally tilted about 15° from the vertical. The maximum angle was set to 40° because if the irradiation angle exceeds 40°, the energy of the laser cannot penetrate deeply into the inflammation and is transferred to the implant. The wavelength selected for the laser was 630 nm, and the diameter was 0.4 mm. Additionally, the laser irradiation location was set to *x* = 12.2 mm, *y* = 0, and *z* = 9 mm, which was the center of the uppermost part of the inflammation.

**TABLE 2:** Parameters of numerical analyses.

Parameter	Range	Number	Remarks
Laser power (*P*_*l*_)	0.0–4.0 W	101	0.04 W steps
Laser irradiation angle (*θ*)	15°–40°	7	5° steps
Laser irradiation time (*t*_*l*_)	100–500 s	5	100 s steps

### 2.4 Validation of Numerical Model

**FIGURE 2:**
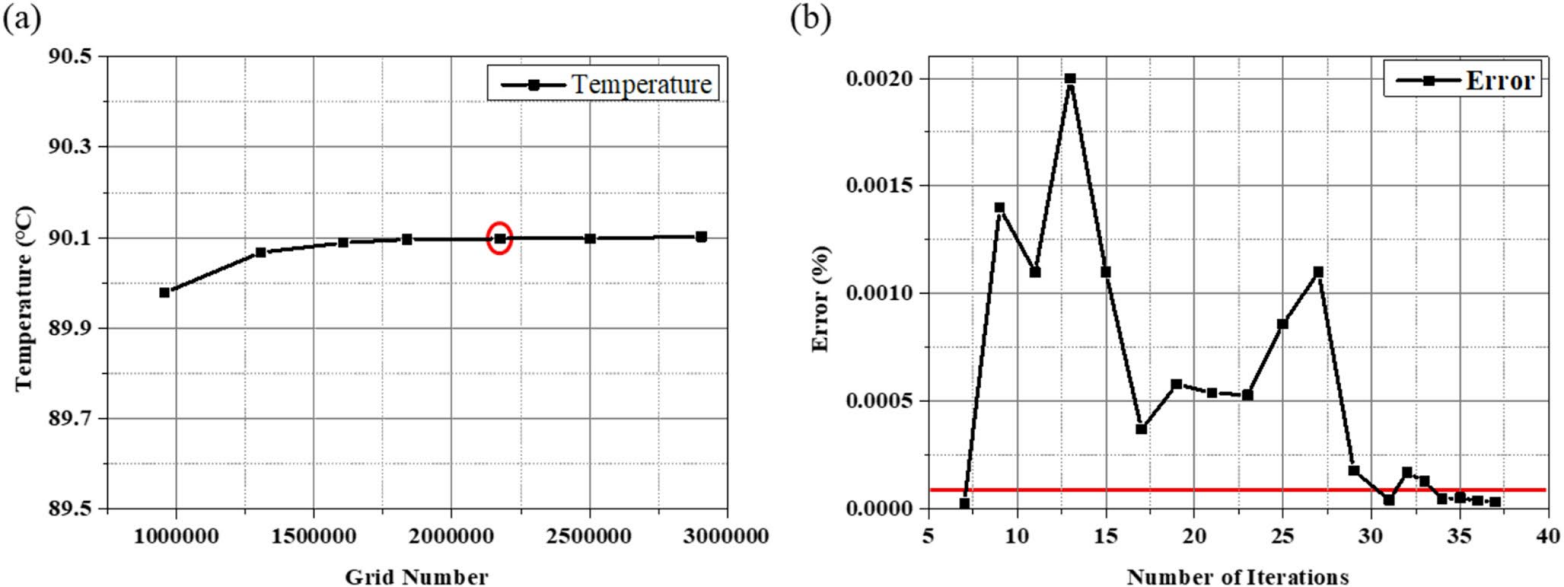
Grid independence test and iteration error results.

In this study, temperature distribution was calculated using COMSOL Multiphysics, based on the finite element method. To verify the validity of the numerical analysis model established in this study, the error was calculated using a grid independence test and iterations, as shown in Figure 2. In the grid independence test, the change in temperature converged to 10^−3^°C or less when the number of grids was approximately 2.3 million. Therefore, the number of grids was set to this value. Furthermore, error as a function of the number of iterations converged to less than 10^−4^% from 33 iterations onward, validating the effectiveness of the numerical modeling.

## 3 Results and Discussion

### 3.1 Determination of Inflammation Alleviation With Various Laser Irradiation Angles and Durations

**FIGURE 3:**
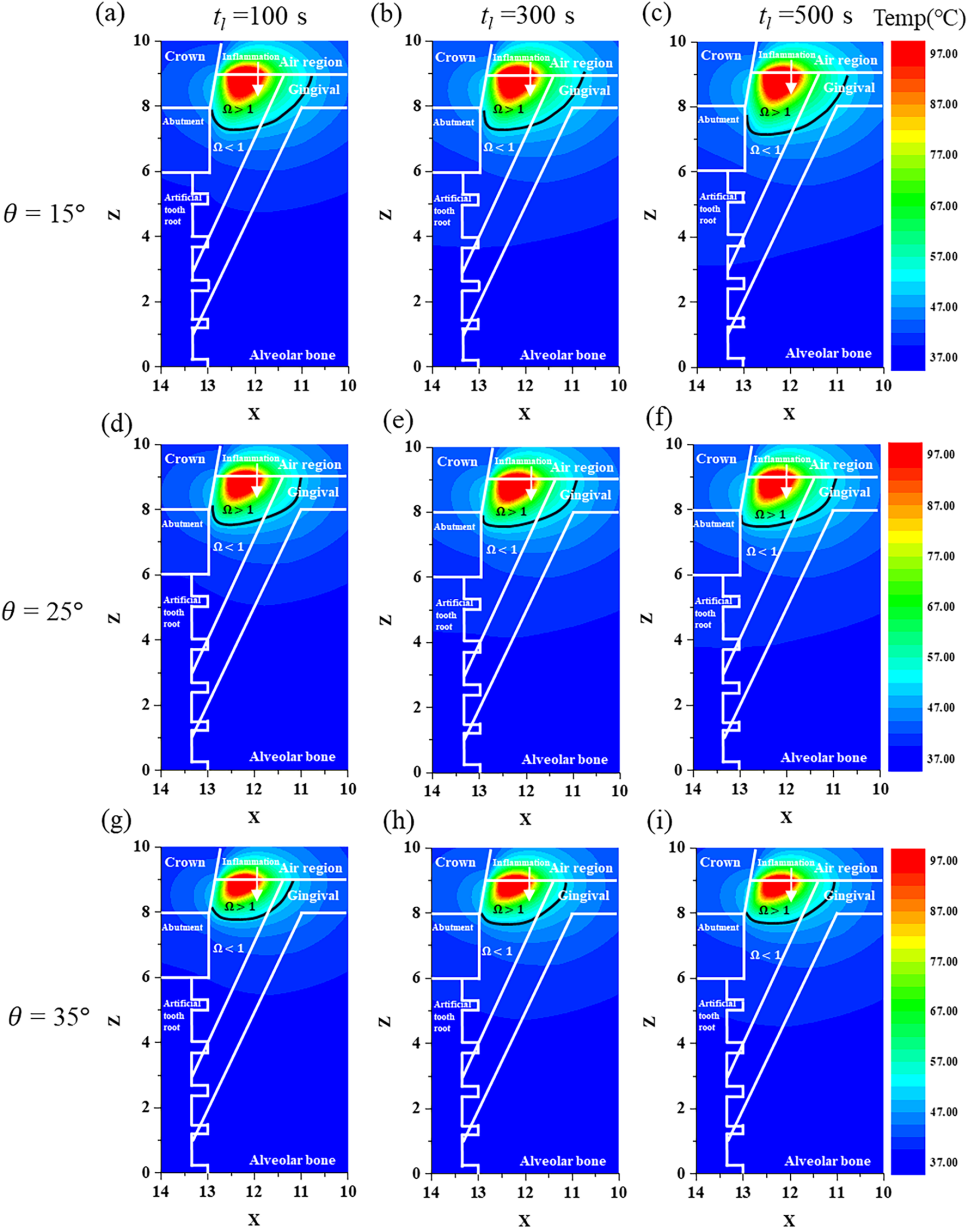
Temperature and irreversible damage distributions for various values of *θ* and *t*_*l*_ (*P*_*l*_ = 2.0 W, XZ plane (*y* = 0)). (a) *θ* = 15°, *t_l_* = 100 s, (b) *θ* = 15°, *t_l_* = 300 s, (c) *θ* = 15°, *t_l_* = 500 s, (d) *θ* = 25°, *t_l_* = 100 s, (e) *θ* = 25°, *t_l_* = 300 s, (f) *θ* = 25°, *t_l_* = 500 s, (g) *θ* = 35°, *t_l_* = 100 s, (h) *θ* = 35°, *t_l_* = 300 s, (i) *θ* = 35°, *t_l_* = 500 s.

First, after fixing *P*_*l*_ at 2.0 W, the temperature distributions and degrees of death of the inflammation and surrounding tissues using various values for *θ* and *t*_*l*_ are shown in Figure 3. In this study, results for temperature are shown in the XZ plane because they are symmetric about the *y*‐axis. The black line shown in each figure represents the point where the Ω is one. If Ω was greater than one, it was determined that irreversible damage had occurred. As shown in Figure 3, greater irreversible alleviation of the inflammation occurred with increasing *t*_*l*_ when *θ* was the same. This was because, as *t*_*l*_ increased, more heat was applied. Additionally, when *θ* increased, the volume range where Ω was greater than one decreased. This was due to the energy density per unit area absorbed by the inflammation decreasing as *θ* increased. This is detailed by comparing the results when *θ* was 15° and 35°. For a *θ* value of 15° (Figure 3a–c), a larger area of inflammation was eliminated as *t*_*l*_ increased. However, for a *θ* value of 35° (Figure 3g–i), death only occurred to a depth of approximately 1 mm from the top of the inflammation, even with a *t*_*l*_ value of 500 s. Conversely, as the depth of the inflammation alleviation increased, death also occurred in normal tissues, which must be considered and will be discussed later.

### 3.2 Determination of Inflammation Alleviation With Various Laser Irradiation Angles and Power Values

**FIGURE 4:**
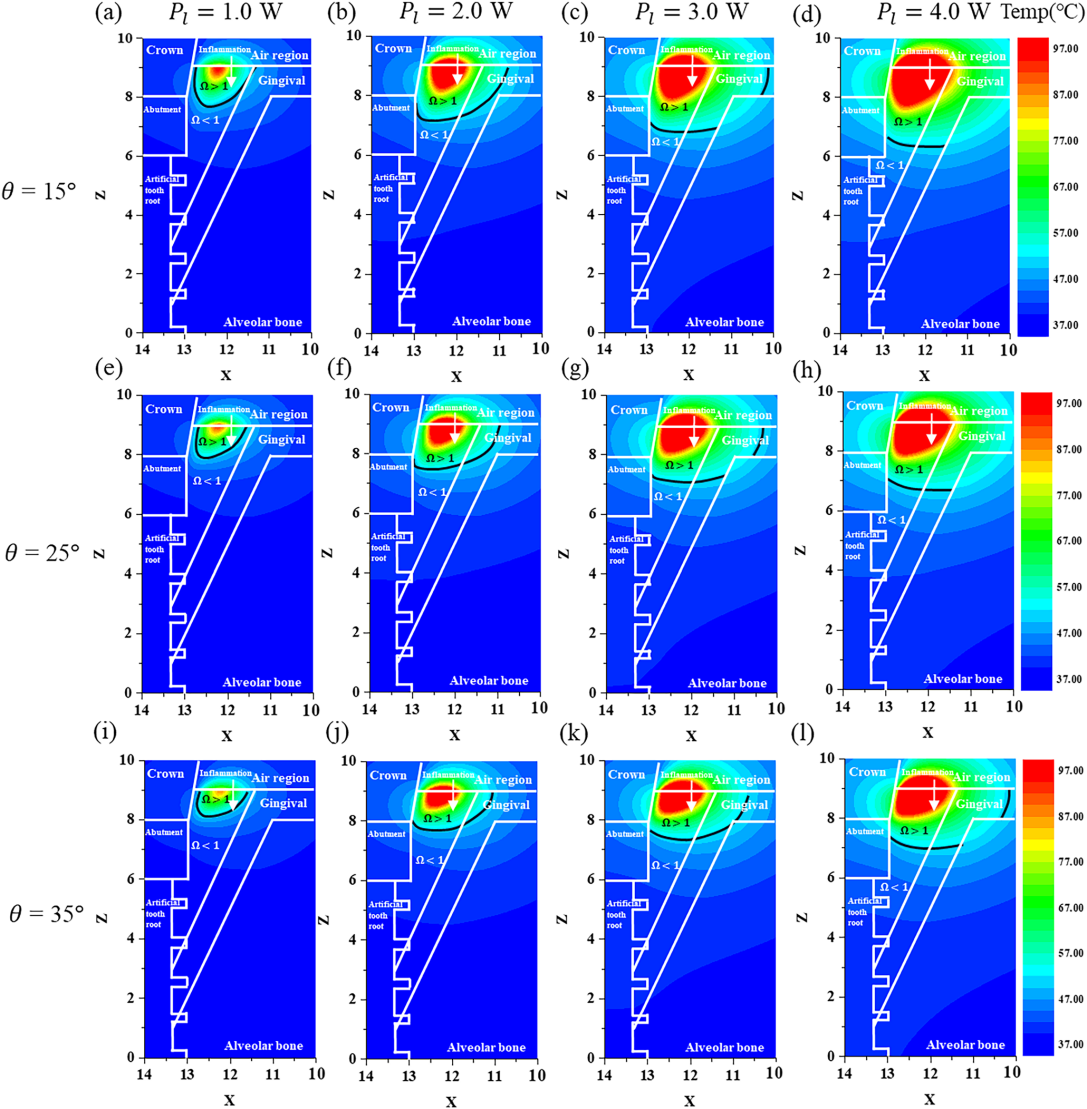
Temperature and irreversible damage distributions with various values of *θ* and *P*_*l*_ (*t*_*l*_ = 500 s, XZ plane (*y* = 0)). (a) *θ* = 15°, *P_l_* = 1.0 W, (b) *θ* = 15°, *P_l_* = 2.0 W, (c) *θ* = 15°, *P_l_* = 3.0 W, (d) *θ* = 15°, *P_l_* = 4.0 W, (e) *θ* = 25°, *P_l_* = 1.0 W, (f) *θ* = 25°, *P_l_* = 2.0 W, (g) *θ* = 25°, *P_l_* = 3.0 W, (h) *θ* = 25°, *P_l_* = 4.0 W, (i) *θ* = 35°, *P_l_* = 1.0 W, (j) *θ* = 35°, *P_l_* = 2.0 W, (k) *θ* = 35°, *P_l_* = 3.0 W, (l) *θ* = 35°, *P_l_* = 4.0 W.

Next, after fixing *t*_*l*_ at 500 s, the temperature distributions and degrees of death of the inflammation and surrounding tissues for various values of *θ* and *P*_*l*_ were determined and are shown in Figure 4. From the results, it can be seen that the percentage increase in the range of inflammation eliminated with increasing *P*_*l*_ was greater for a small *θ* than for a large *θ*. This is evident in the cases of 15° (Figure 4a–d) and 35° (Figure 4i–l). The reason for this is that the energy density per unit area absorbed by the inflammation was greater when *θ* was 15° compared to when *θ* was 35°.

### 3.3 Arrhenius Variable Analysis of Normal Tissue and Inflammation

In the previous sections, the degree of irreversible alleviation of the inflammation and the temperature distribution in the surrounding tissues were determined for various values of *P*_*l*_, *θ*, and *t*_*l*_. This section discusses the analysis results for *ϕ* ^*N*^_*Arrh*_ and *ϕ*_*Arrh*_ as *t*_*l*_ increased at each *θ*.

**FIGURE 5:**
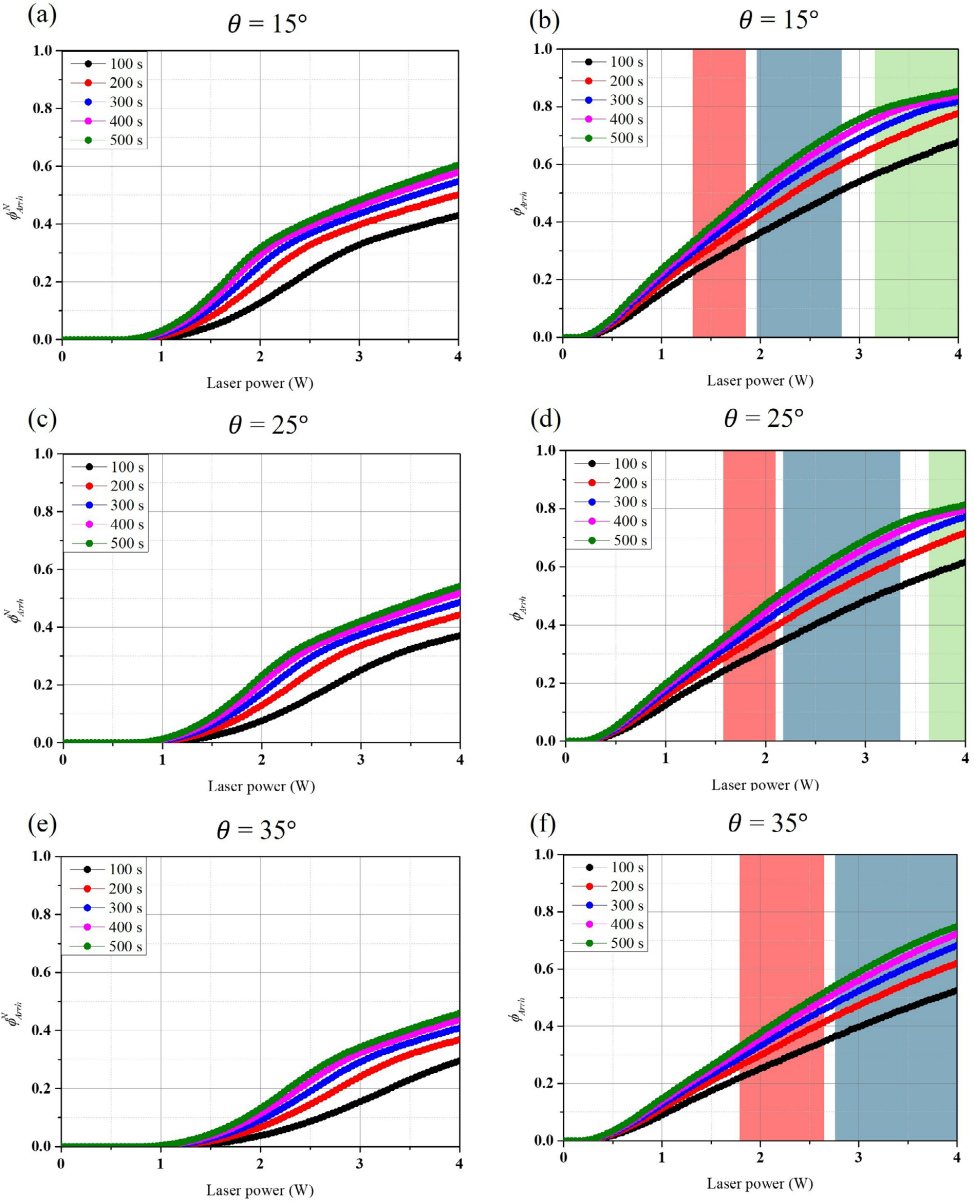
Normal tissue Arrhenius thermal damage ratios and Arrhenius thermal damage ratios for various irradiation times at each irradiation angle (red area: *ϕ* ^*N*^_*Arrh*_ = 0.1, blue area: *ϕ* ^*N*^_*Arrh*_ = 0.3, green area: *ϕ* ^*N*^_*Arrh*_ = 0.5). (a) *θ* = 15°, *ϕ^N^_Arrh_*, (a) *θ* = 15°, *ϕ_Arrh_*, (a) *θ* = 25°, *ϕ^N^_Arrh_*, (a) *θ* = 25°, *ϕ_Arrh_*, (a) *θ* = 35°, *ϕ^N^_Arrh_*, (a) *θ* = 35°, *ϕ_Arrh_*.

Figure 5 show *ϕ* ^*N*^_*Arrh*_ and *ϕ*_*Arrh*_ as a function of *t*_*l*_ for different *θ*. As expected, the trend of *ϕ* ^*N*^_*Arrh*_ shows that it increased with increasing *t*_*l*_ and decreased with increasing *θ* as a result of a decrease in energy density. It can be seen that *ϕ* ^*N*^_*Arrh*_ did not vary linearly with increasing *P*_*l*_ for all *θ* and *t*_*l*_.

The trend for *ϕ*_*Arrh*_ was more interesting. When analyzing the results for *ϕ*_*Arrh*_, in order to simultaneously see the degree of *ϕ* ^*N*^_*Arrh*_, which could be considered damage to normal tissue, the ranges of *P*_*l*_ where *ϕ* ^*N*^_*Arrh*_ was 0.1, 0.3, and 0.5 were indicated as red, blue, and green regions, respectively. *ϕ*_*Arrh*_, which indicates irreversible alleviation of the inflammation, tended to increase with increasing *t*_*l*_ and *P*_*l*_, and decrease with increasing *θ*. These results indicate that at the same *t*_*l*_ and *P*_*l*_, a smaller *θ* or a more concentrated *P*_*l*_ in a narrow area resulted in a greater *ϕ*_*Arrh*_. However, the maximum *ϕ*_*Arrh*_ that could be obtained within a certain range of *ϕ* ^*N*^_*Arrh*_ differed. For example, in the red region (*ϕ* ^*N*^_*Arrh*_ = 0.1), the maximum value of *ϕ*_*Arrh*_ that could be obtained while maintaining normal tissue damage within 10% was 0.351, as seen in Figure 5f, where *θ* is 35°. Figure 5f shows that within the red region with *ϕ* ^*N*^_*Arrh*_ = 0.1, *P*_*l*_ could be increased to 2.64 W, resulting in a higher *ϕ*_*Arrh*_ than in the cases of Figure 5b,d. However, when *θ* became 40°, the maximum value of *P*_*l*_ increased to 2.92 W, but the maximum value of *ϕ*_*Arrh*_ decreased to 0.342. This was because a higher *P*_*l*_ was required as a result of the decrease in energy density with increasing *θ*, but the range of temperature rise in the inflammation was reduced due to the decrease in the absorption range of the laser energy. Similarly, the blue region with *ϕ* ^*N*^_*Arrh*_ = 0.3 shows a higher *ϕ*_*Arrh*_ value in Figure 5f, where *θ* = 35°, which allowed *P*_*l*_ to be increased to 4 W. However, the maximum value of *ϕ*_*Arrh*_ decreased when *θ* reached 40°. Additionally, *ϕ* ^*N*^_*Arrh*_ did not reach 0.5 when *θ* exceeded 30°. Table 3 summarizes the range of laser intensity required for each laser angle according to *ϕ* ^*N*^_*Arrh*_ and the corresponding range of *ϕ*_*Arrh*_ under the overall numerical analysis conditions presented in this study. To maximize the alleviation of inflammation while minimizing damage to normal tissues, irradiating a wider area was more advantageous than increasing the energy density of the laser. Furthermore, the reason for the higher laser power in this study than in conventional laser therapy is due to the shape of the inflammation. Since the shape of the inflammation is longitudinally elongated, it is necessary to increase the laser power in order to cause thermal damage to the lower part of the inflammation.

**TABLE 3:** Required laser intensity and ranges of *ϕ*_*Arrh*_ results depending on *ϕ* ^*N*^_*Arrh*_.

Laser irradiation angle (*θ*)	Range	*ϕ* ^*N*^_*Arrh*_					
		0.1		0.3		0.5	
		Min.	Max.	Min.	Max.	Min.	Max.
15°	*P*_*l*_ [W]	1.36	1.88	1.96	2.84	3.16	> 4.00
	*ϕ*_*Arrh*_	0.340	0.343	0.514	0.523	0.678	0.782
20°	*P*_*l*_ [W]	1.44	2.00	2.08	3.04	3.36	> 4.00
	*ϕ*_*Arrh*_	0.342	0.348	0.524	0.527	0.653	0.785
25°	*P*_*l*_ [W]	1.56	2.20	2.28	3.32	3.64	> 4.00
	*ϕ*_*Arrh*_	0.345	0.350	0.530	0.539	0.616	0.753
30°	*P*_*l*_ [W]	1.68	2.40	2.48	3.68	> 4.00	—
	*ϕ*_*Arrh*_	0.351	0.345	0.534	0.538	> 0.574	—
35°	*P*_*l*_ [W]	1.88	2.64	2.76	> 4.00	> 4.00	—
	*ϕ*_*Arrh*_	0.346	0.351	0.525	0.542	> 0.525	—
40°	*P*_*l*_ [W]	2.08	2.92	3.08	> 4.00	> 4.00	—
	*ϕ*_*Arrh*_	0.338	0.342	0.471	0.539	> 0.471	—

## 4 Conclusion

In this study, the effects of laser irradiation angle on photothermal therapy outcomes for peri‐implantitis were numerically analyzed under varying laser irradiation times and intensities. Temperature distributions in the inflamed and surrounding tissues were calculated using the Pennes bioheat equation, and irreversible alleviation of inflammation and damage to normal tissues were analyzed using the Arrhenius damage integral. The study determined the effective range of laser intensity and the extent of inflammation alleviation based on *ϕ* ^*N*^_*Arrh*_ across all numerical conditions. In conclusion, increasing the irradiation angle and covering a wider area is advantageous for maximizing inflammation alleviation while minimizing damage to normal tissues. However, confirming that excessively increasing the laser irradiation angle reduced the range of temperature increase within the inflammation area due to decreased absorption of laser energy, thereby reducing the degree of inflammation alleviation. Based on these results, further studies are expected to determine the range of laser intensity corresponding to *ϕ* ^*N*^_*Arrh*_ values of 0.1, 0.3, and 0.5. Finally, the best therapeutic effect was confirmed when the laser angle was 15°, and the range of laser power to satisfy *ϕ* ^*N*^_*Arrh*_ of 0.1, 0.3, and 0.5 was 1.36–1.88 W, 1.96–2.84 W, and 3.16 W or more, respectively. This will help identify the degree of inflammation alleviation under different conditions, facilitating the suggestion of practical treatment methods for clinical applications. However, the Arrhenius integral value used in this study is a fixed value, which may be difficult to cover a wide temperature range. Therefore, in the future, we believe that applying different values for each temperature range will provide more accurate results. Lastly, since this study analyzed the results through numerical analysis, it is considered necessary to verify the results of this study through experiments in order to apply the results of this study to practical applications.

**Nomenclature**
A
: frequency factor (1/s)
*c*_*p*_
: specific heat (J/(kg·K))
*E*_*a*_
: activation energy (J/mol)
k
: thermal conductivity (W/(m·K))
*n*_*1*_
: refractive index of air
*n*_*2*_
: refractive index of inflammation
*P*_*l*_
: laser power (W)
q
: volumetric heat source (W/m^3^)
*r*_*l*_
: laser radius (mm)
*R*_*1*_
: specular reflection value
*R*_*2*_
: diffuse reflection value
*R*_*t*_
: total reflectivity
R
: ideal gas constant (J/(mol·K))
T
: temperature (K)
t
: time (s)

**Greek symbols**
𝜃: Irradiation angle (°)
*μ*
: optical coefficient (1/cm)
*μ’*
: reduced Optical coefficient (1/cm)
𝜌: Density (kg/m^3^)
*ϕ*_*Arrh*_
: Arrhenius thermal damage ratio
*ϕ* ^*N*^_*Arrh*_
: normal tissue Arrhenius thermal damage ratio
Ω: Arrhenius damage integral value
*ω*_*b*_
: blood perfusion rate (1/s)

**Subscripts**
a
: absorption
b
: blood
l
: laser
m
: metabolic
s
: scattering
tot
: attenuation
x, y, z
: notation of direction

## Author Contributions

Conceptualization: D.K., H.‐S.K., and H.K. Data curation: D.K. Formal analysis: D.K. Funding acquisition: H.K. Investigation: D.K. Methodology: D.K. Project administration: H.‐S.K. and H.K. Resources: H.K. Software: D.K. Supervision: H.‐S.K., H.K. Validation: D.K. Visualization: D.K. Writing – original draft: D.K. Writing – review and editing: D.K., H.K. All the authors have read and approved the final version of the manuscript.

## Ethics Statement

The authors have nothing to report.

## Conflicts of Interest

The authors declare no conflicts of interest.

## Data Availability

The data that support the findings of this study are available from the corresponding author upon reasonable request.
